# Automated ex-situ detection of pitting corrosion and its effect on the mechanical integrity of rare earth magnesium alloy - WE43

**DOI:** 10.1016/j.bioactmat.2021.06.024

**Published:** 2021-07-07

**Authors:** Kerstin van Gaalen, Felix Gremse, Felix Benn, Peter E. McHugh, Alexander Kopp, Ted J. Vaughan

**Affiliations:** a Biomechanics Research Centre (BioMEC), Biomedical Engineering, School of Engineering, College of Science and Engineering, National University of Ireland Galway, Galway, Ireland; b Meotec GmbH, Aachen, Germany; c Institute for Experimental Molecular Imaging, RWTH Aachen University, Aachen, Germany; d School of Mechanical and Aerospace Engineering, Queen's University Belfast, Belfast, United Kingdom

**Keywords:** Magnesium, Corrosion, Degradation, Micro-computer-tomography, Pit detection, Automatization, Mechanical integrity

## Abstract

This study develops a three-dimensional automated detection framework (*PitScan*) that systematically evaluates the severity and phenomenology of pitting corrosion. This framework uses a python-based algorithm to analyse microcomputer-tomography scans (μCT) of cylindrical specimens undergoing corrosion. The approach systematically identifies several surface-based corrosion features, enabling full spatial characterisation of pitting parameters, including pit density, pit size, pit depth as well as pitting factor according to ASTM G46-94. Furthermore, it is used to evaluate pitting formation in tensile specimens of a Rare Earth Magnesium alloy undergoing corrosion, and relationships between key pitting parameters and mechanical performance are established. Results demonstrated that several of the parameters described in ASTM G46-94, including pit number, pit density and pitting factor, showed little correlation to mechanical performance. However, this study did identify that other parameters showed strong correlations with the ultimate tensile strength and these tended to be directly linked to the reduction of the cross-sectional area of the specimen. Specifically, our results indicate, that parameters directly linked to the loss of the cross-sectional area (e.g. minimum material width), are parameters that are most suited to provide an indication of a specimen's mechanical performance. The automated detection framework developed in this study has the potential to provide a basis to standardise measurements of pitting corrosion across a range of metals and future prediction of mechanical strength over degradation time.

## Highlights

• In-vitro immersion study of dog bones manufactured from a WE43 Magnesium alloy.
• Novel approach characterizing spatial pit formation using micro-CT scans.
• Comparison of mass loss by hydrogen gas measurement and volume loss by μCT scans.
• Correlation between mechanical strength and geometrical pit formation features.

## Graphical abstract

**Figure:**
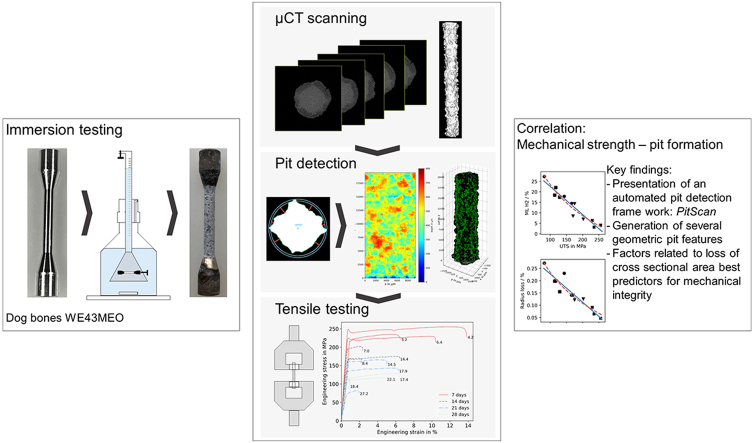

## 1 Introduction

Magnesium alloys show great potential as biodegradable alternatives to permanent metallic orthopaedic implants as they are osteo-inductive [[1], [2], [3], [4], [5], [6]] and their mechanical properties are comparable to native bone, thereby avoiding stress-shielding complications raised by traditional metallic implants [7]. Magnesium-based implants could eliminate the need for second surgeries for implant removal, thus reducing additional trauma and recovery time to the patient. However, rapid degradation behaviour has meant that magnesium-based implants have been unable to fulfil load-bearing requirements for the duration of the tissue healing process [8,9].

Magnesium alloys undergo degradation through a range surface-based corrosion mechanism, including galvanic, pitting, intergranular corrosion in physiological conditions [10]. Galvanic corrosion results in a protective oxide layer formation on the implant surface. The surrounding presence of chloride ions is able to break down this partly protective oxide layer, leading to an ongoing transformation of bulk material to oxide layer. A theoretical uniform corrosion is practically non-existent for light weight metal alloys, as microstructural inhomogeneities and impurities are introduced during the manufacturing process. Consequently, non-uniform corrosion phenomena such as pitting corrosion subsequently takes place on the material surface [[11], [12], [13], [14], [15], [16]]. Pitting corrosion, which describes the locally varying corrosion rate, induces high variation of mechanical integrity of medical implants, which has limited their implementation in load-bearing applications [17,18]. Recent efforts to improve performance have sought to control the corrosion rate of magnesium by varying the alloy composition or through the application of protective surface coatings [10,[19], [20], [21], [22]]. With these, and the vast majority of other in vitro studies of magnesium, bulk measurements of corrosion are generally considered, whereby corrosion rates are determined through techniques such as electrochemical tests [[23], [24], [25], [26]], hydrogen evolution [17,18,21,25,[27], [28], [29], [30], [31]] or mass/volume loss measurements [[32], [33], [34]]. However, these studies provide limited information on how localised corrosion affects mechanical performance. Recent studies have quantified the non-uniform relationship between bulk mass loss and mechanical strength of both AZ31 and WE43 magnesium alloys undergoing corrosion [17,18]. While the disproportionate reduction in load-bearing capacity of Magnesium alloys, compared to corresponding mass loss, is attributed to the evolution of pitting corrosion observed across specimens, there remains little quantitative understanding on how pit formation (e.g. severity and spatial distribution) affects overall mechanical performance with other studies generally examining pitting corrosion in magnesium through largely qualitative approaches [15,26,35].

Pitting corrosion affects a wide range of metals and is a critical aspect of environmental degradation of components in other structural applications, including marine and aerospace. ASTM G46-94 provides the standard guide for the examination and evaluation of metals undergoing pitting corrosion [36], whereby the severity of pitting is established through metallography and visual analysis. Here, material surfaces are examined two-dimensionally and standard ratings for pitting may be expressed in terms of the pit density, pit size or pit depth. The degree of metal penetration may also be expressed in terms of a pitting factor, which is the ratio defined as the deepest surface penetration depth divided by the average depth. However, only a few studies have examined magnesium alloys using the parameters outlined in this standard guide [15,37] and there are still several limitations with the approaches. Firstly, there is no established methodology available that can systematically evaluate these parameters, with current techniques using cross-sectional microscopy or surface examination through profilometry to determine pitting metrics. The main disadvantage with these approaches is that they are two-dimensional and do not consider the entirety of the specimen. Furthermore, they require destructive processing, and a major difficulty is that much of material will be actually removed by polishing and cannot be analysed [38]. Secondly, there is little or no quantitative understanding as to how pit density, pit size, pit depth and/or pitting factor relate to the mechanical integrity of the specimen. To advance our understanding of pitting corrosion, it is critical that standardised detection methods are established to measure key pitting parameters and their effect on the load-bearing integrity of structure determined through concurrent mechanical testing. To date, only a limited number of studies have proposed methods to automatically track pitting corrosion in metals, but these have never been applied to magnesium [[39], [40], [41], [42]].

The objective of this study is to develop a three-dimensional automated detection framework that systematically evaluates the severity and phenomenology of corrosion and relationships between key pitting parameters and mechanical performance are established. This detection framework (from now on called *PitScan*) uses a python-based algorithm to analyse microcomputer-tomography scans (μCT) of cylindrical specimens undergoing corrosion. The approach systematically identifies several pitting features on the corroding surface, enabling full geometric characterisation of pitting parameters, including pit density, pit size, pit depth and pitting factor. Within this study we use pitting corrosion as a term unifying all localised surfaced based corrosion effects (e.g. pitting corrosion, intergranular) [10].

## 2 Material and methods

**Fig. 1:**
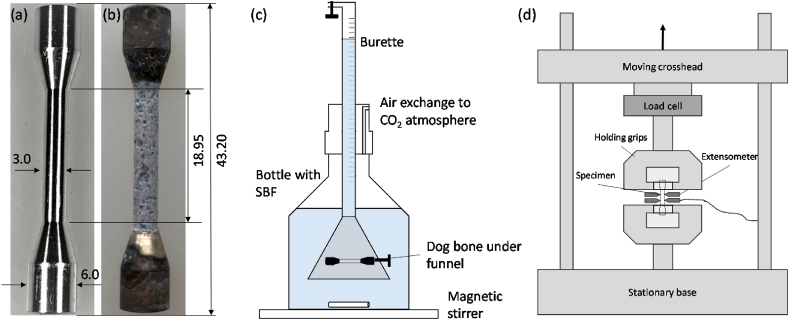
Dimensions of the Magnesium WE43 test specimens that were turned from a 6.5 mm cylindrical rod a) undegraded sample and b) following 14 days immersion; c) Schematic overview of immersion test setup [21]; d) Schematic overview of tensile test.

Cylindrical dog-bone test specimens were produced from a chill casted extruded Magnesium WE43MEO alloy that had a nominal composition of 1.4–4.2% Y, 2.5–3.5% Nd, <1% (Al, Fe, Cu, Ni, Mn, Zn, Zr) and balance Mg (in wt%) (Meotec GmbH, Germany). The cast material underwent an extrusion process to form 6.5 mm rods, which was followed by a turning process that produced cylindrical dog-bone sample whose dimensions are shown in Fig. 1(a). Inductively coupled plasma atomic emission spectroscopy (ISC-OES) measurements confirmed the chemical composition according to the manufacture's specification. Immersion testing was performed for 28 days to induce pitting-based corrosion in the sample, as shown in Fig. 1(b). At weekly time-points, micro-computed tomography (CT) scanning and mechanical testing of corroded samples were carried out. Correlations were established between mechanical performance and the geometrical pit formation using a novel pit detection algorithm that describes the pit formation of the corroding magnesium rods.

### 2.1 Immersion testing

Immersion testing followed a similar protocol to that described by Kopp et al. [21] and is represented schematically in Fig. 1(c). Samples were placed in the bottle by mounting them under a silicone funnel that was fixed to the glass burette. Bottles were placed on a magnetic stirrer to ensure a homogenous pH level in the solution. Here a conventional-Simulated Body Fluid (c-SBF) was used [43], whose composition is provided in Table 1. Each bottle was filled with 600 mL, which leads to a volume to sample surface ratio (V/S) of 3.36 mL/mm^2^, which is more than 10 times greater than the ratio suggested in the standard (minimum of 0.20 mL/mm^2^) [44]. Clamping areas were protected with chemically inert polyolefin shrinking hose to ensure that these areas are not degrading.

**Table 1:** Chemical compositions of c-SBF in 1 L pure water [43].

Reagents	in 1 L
NaCl	8.035 g
NaHCO_3_	0.355 g
KCl	0.225 g
K_2_HPO_4_	0.176 g
MgCl_2_	0.145 g
CaCl_2_	0.292 g
Na_2_SO_4_	0.072 g
Tris puffer pH 7.5 (1 mol)	50 mL

Separate sample groups were immersed for periods of 7, 14, 21 and 28 days, with n = 3 per group. Tests were carried out in a humidified incubator (HERAcell 150i, Thermo Fischer Scientific Inc., Waltham, USA) at 37 ± 1 °C, under an atmosphere with 5% CO_2_. Hydrogen gas measurement is a widely used method tracking mass loss for Magnesium and its alloys in in-vitro immersion test-setups [45]. The evolved hydrogen gas (H_2_) was captured in the burette and tracked by an eudiometer. Mass loss (ML) was derived from hydrogen gas evolution based on the cathodic reaction equation describing the corrosion process [45], given by,

(1) $$2H_{2}O+2e^{-}\to 2OH^{-}+H_{2}$$

The corresponding anodic reaction of magnesium corrosion is,

(2) $$Mg\to Mg^{2+}+2e^{-}$$

So the overall reaction is described by,

(3) $$Mg+2H_{2}O\to Mg{\left(OH\right)}_{2}+H_{2}$$

The degradation layer (passive layer) mostly comprises of magnesium hydroxide (Mg(OH)_2_). In the presence of chloride ions, like in body fluids, this layer can be destroyed by the formation of magnesium chloride.

(4) $$Mg{\left(OH\right)}_{2}+2Cl^{-}\to MgCl_{2}+2OH^{-}$$

Because magnesium chloride has a greater solubility in water, local corrosion (pitting) occurs in these areas [46]. Additionally, the corrosion rate can be calculated as described in ASTMG31 - 12a [44], according to the following equation that represents corrosion rate in mm/year:

(5) $$CR=\;\frac{8.76\;10^{4}W}{At\rho }=\frac{8.76\;10^{4}V_{H_{2}}\rho_{H_{2}}M_{Mg}}{At\rho_{Mg}M_{H_{2}}}$$

where $V_{H_{2}}$ is the hydrogen gas volume in mL and ρ is the density in g/cm³. M is the molecular weight in g/mol, A is the exposed surface area in cm^2^, and t is the overall immersion time in hours. After removal from the c-SBF immersion media, samples were immediately dried to ensure the corrosion process had stopped. Then pH of each solution media was measured with a pH meter (Sartorius PB-11, Satorius AG, Göttingen, Germany).

### 2.2 Micro computed tomography

**Fig. 2:**
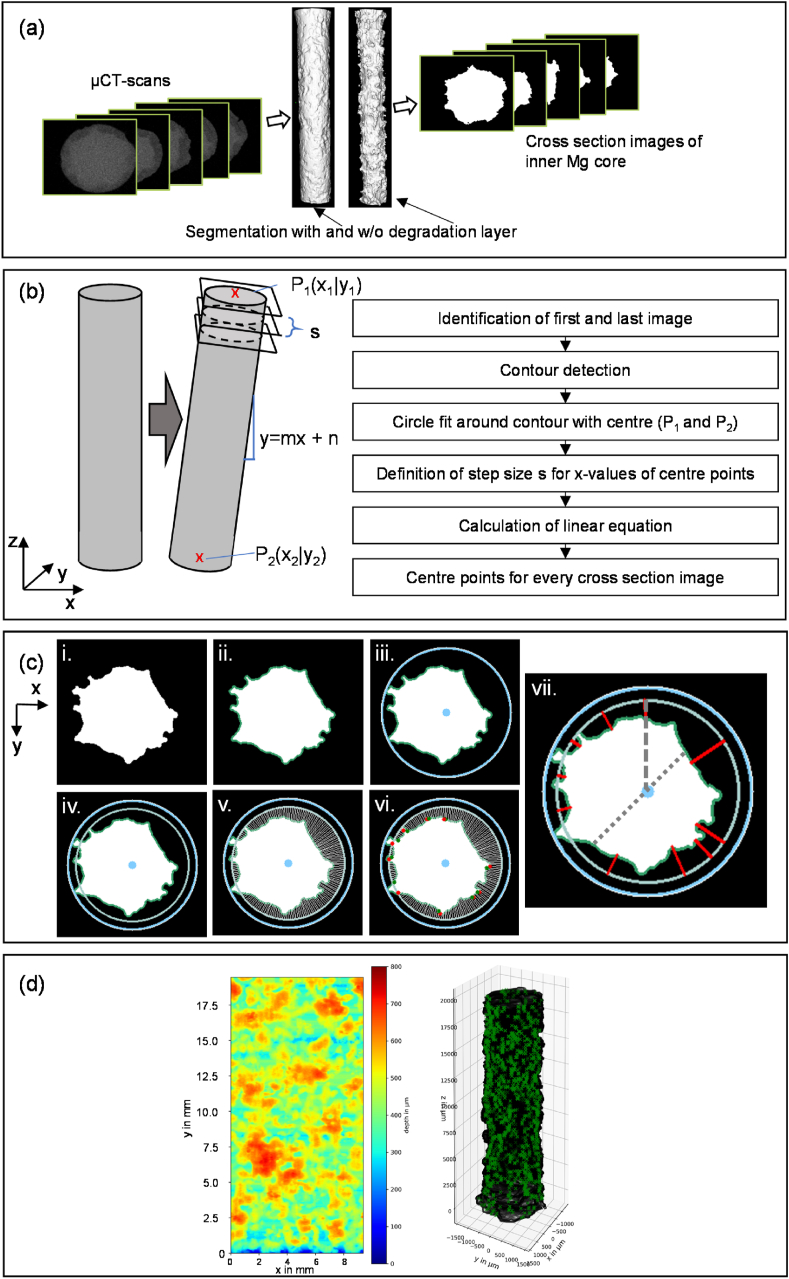
Basic principle of PitScan (a) from μCT cross section images to the segmentation of inner Mg core to black and white cross section images of Mg core; (b) Basic principle of the correction function to correct oblique positions during the scanning procedure. Left: perfect straight sample; middle: Oblique positioned sample; Right: description of basic algorithm of the centre point detection chain (c) Automated image recognition process chain i. Raw input, ii. Contour detection, iii. Circle plotting, iv. Material portion, v. Depth tracking, vi. Pit on-off tracking, vii. Determination of deepest point of each pit. Grey dashed line fitted radius, grey dotted line minimum Mg core width; (d) Output images: left: Heatplot of the surface of the cylinder, right: total 3D reconstruction of the gauge length, every green cross marks the deepest point of one pit. (For interpretation of the references to colour in this figure legend, the reader is referred to the Web version of this article.)

Following sample immersion, μCT scans of all dog bone specimens were performed (Skyscan 1272, Bruker, Belgium). Samples were scanned with X-ray emission parameters of 100 kV and 100 μA, which provided a pixel size of 15 μm and enabled segmentation of both the inner magnesium core and the degradation layer of each cylindrical sample. The work here focuses on the magnesium core, as it is assumed that the degradation layer does not contribute to the load-bearing capacity of the specimen. Imalytics Preclinical Software (Gremse‐IT GmbH, Germany) [47] in combination with ImageJ (version 1.52, Wayne Rasband, National Institutes of Health, Bethesda, MD, USA) was used to generate binarized images of the inner magnesium core, with the process shown schematically in Fig. 2(a). Firstly, a Gaussian filter was applied to the raw input stack and the inner magnesium core was identified by manually adjusting the threshold for the brighter part of the specimen. With Imalytics software, the total volume of the remaining core was calculated and the corresponding volume loss (VL) of the gauge length of each dog-bone specimen was determined. With equation (5) the corrosion rate was determined, and the weight loss was calculated with $W\;=\;\Delta VL_{Mg}\rho_{Mg}$. Subsequently, the segmentation file was imported into ImageJ to receive the single cross section images from the stack, with the inner core existing only of white pixels and the remaining part of black ones (binarization).

### 2.3 Mechanical testing

Following micro-CT scanning, uniaxial tensile testing of the cylindrical dog bone specimens was carried out at a constant velocity of 1.0 mm/min until failure (10 kN load cell, ZwickRoell GmbH & Co. KG, Germany). Displacement of the gauge length was tracked through extensometers (makroXtens, ZwickRoell GmbH & Co. KG, Germany) attached at the surface (see Fig. 1(d)). In all cases, the initial cross section area of the gauge section (A = πr^2^ = π1.5^2^ mm^2^ = 7.07 mm^2^) was used to determine the nominal stress.

### 2.4 Pit detection

The algorithms developed within this study for “*PitScan”,* enable a standardised detection of the three-dimensional degradation formation in the cylindrical magnesium specimens through automated image recognition of micro-CT scans. *PitScan* analyses a stack of two-dimensional images of the material cross section, derived from the micro-CT scan, within an automated process chain of image recognition implemented in Python with OpenCV. *PitScan* uses the binarized images of the solid inner magnesium core and starts with radial contouring of this core with a subsequent pit detection. All this is individually implemented for each layer and then a three-dimensional reconstruction of the pitting formation is performed.

Firstly, radial contouring of each binarized image is carried out. The complete tracking is fundamentally based on the correct definition of the initial (before degradation) centre point of the sample. To exclude the influence of slightly oblique positioned samples in the scanner, the first and the last image is taken to generate a linear correction equation (see Fig. 2(b)). These two images are processed by the following steps: First, the outer contour is detected (see Fig. 2(c)ii). Then a circle is fitted around this contour, which gives the coordinates of the centre points. Consequently, two points are identified $P_{1}=\left(x_{1}|y_{1}\right)$ and $P_{2}=\left(x_{2}|y_{2}\right)$. From these two points, a linear equation is computed. To get the centre points of every single image, the step size s for calculating the x-coordinates is defined by:

(6) $$\text{s}=\frac{{\text{x}}_{2}-{\text{x}}_{1}}{\text{amount of images}}$$

So ${\text{x}}_{\text{i}}$ can be calculated by:

(7) $${\text{x}}_{i}={\text{ x}}_{1}+\left(i*s\right)$$

The corresponding y-coordinates are calculated by:

(8) $${\text{y}}_{i}=\left({\text{x}}_{i}*m\right)\;+\text{ n}$$

After the generation of the centre points, every image runs through the same process chain, depicted in Fig. 2(c). First, a pit tracking in 2D of the images takes place. The process starts with the contour detection of the raw black and white image. Second, a circle is fitted with the radius as the greatest distance from the previous calculated centre point to the contour. In a subsequent step, the fitted radius is reduced to avoid tiny sharp edges having any influence on the fitted radius. Hence, a smoothing takes place and the radius is decreased until a material ratio of 20% is reached (Fig. 2(c)iv) [48]. It indicates the ratio of the circumference of the decreased radius around the contour to the intersections with the contour. This radius $r_{1}$ will be considered for the calculation of the radius loss ($r_{1}/r_{0}$) in every cross section, respectively. We assume that $r_{1}$ is related to the uniform degradation which needs to be subtracted to identify pits. In this study $r_{0}$ is always 1500 μm which is the initial gauge length radius of the tensile test specimen. With this radius, the radial distances to the contour are tracked for every two degrees circumferentially (Fig. 2(c)v). All these values are stored in a one-dimensional array: $d_{i}\;\left(\text{with}\;i\in \;\left\{j\leftarrow N\;|\;j\leq 180\right\}\right)$ that allows the start and end points of an individual pit to be tracked. Full details of this process are described in Algorithm 1. The output array is P, where 1 corresponds to a pit, 0 to no pit.

Algorithm 1 Pit on off definition.

**Figure:**
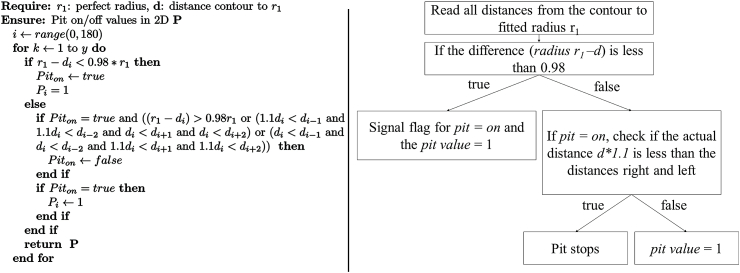

As mentioned, this process automatically runs through all cross-section images (or layers), while the output is stored in one combined array: A (with the number of rows corresponding to the total number of cross section images). Every row has the following information: (i) Array d distance from fitted radius to contour every 2°; (ii) Array P: Pit on (P_i_ = 1) Pit off (P_i_ = 0) value for every 2nd degree and (iii) Fitted radius r_1_.

The tracking in three-dimensions is based on checking whether there are pits at the same range of degrees layer-by-layer. If there is a pit at the same location in two images next to each other the algorithm will “bond” those two as one pit and checks in the next layer to determine if there is also a pit at the same range of degrees, and so on. One main achievement of this process is that the deepest point of a pit can be identified for each 3D pit with its exact position. This principle is further described in Algorithm 2. As input, the array B is taken. B is generated from A, where every row belongs to one pit (P_i_ = 1, with no interruption) with the following information: (i) The layer of the pit; (ii) Range of degrees; and (iii) Distance contour to r_1_.

Algorithm 2 Pit tracking in 3D running through the entities of **P**.

**Figure:**
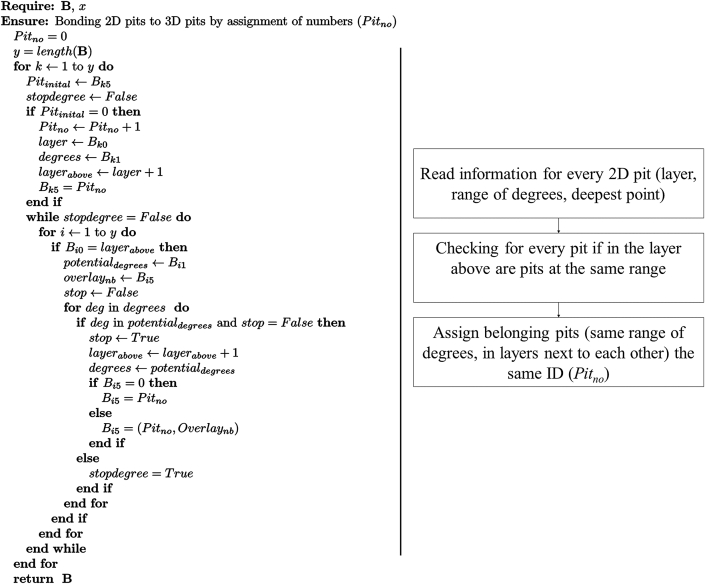

Only pits with a maximum depth greater than 50 μm are considered as a pit. The algorithm provides the following outputs. Firstly, two images are generated: A surface contour plot of pit depth around the cylindrical gauge length (Fig. 2(d) left); and a total three-dimensional reconstruction of the specimen where every green cross marks the deepest point of one tracked pit (Fig. 2(d) right). Second, several characteristic parameters are calculated to get a fully quantified description of the pit formation. All tested dog bone specimens were analysed by this method (described in detail in section 2.5). Verification of the detection process was conducted by considering manual measurements across one specimen from each timepoint. For each one, radial measurements were taken using ImageJ in 2.5 mm distance increments along the longitudinal axis and radial (initial radius to the interface degradation layer - Mg core) and compared with the generated 3D contour plot. This analysis did not show any discrepancies between manual and automated measurements. These results may be found in Appendix A.

### 2.5 Regression fitting

Subsequent to the three-dimensional analysis, several geometric parameters describing pit formation are directly calculated by the pit detection tool. Table 2 outlines all parameters that are calculate, along with a detailed description. For samples undergoing corrosion, correlation between each of these geometric parameters and ultimate tensile strength of the specimen were determined by fitting both linear (y = mx + n) and exponential functions (y = a ∙ e^bx^), with the coefficient of determination was calculated for each fit.

**Table 2:** Detailed description of generated geometrical parameters within the pit detection tool (d: single pit depth, i: number of cross section images, r: fitted radius, r_0_ initial radius).

Parameter	Symbol	Description
Mass loss H_2_ (%)	ML	Mass loss generated by hydrogen evolution during immersion testing
Volume loss from μCT (%)	VL	Volume loss calculated in Imalytics software from μCT scans
No pits	n	Total number of pits
Pits per cm^2^	$\bar{n}$	Tracked pits per cm^2^ (with the average fitted radius):
Volume loss through pits (%)	$VL_{pits}$	Sums up only the volumes of the real pits (d > 50 μm):
Av. Radius loss	$\bar{RL}$	Average of all fitted radii for ever layer: $\bar{RL}=\frac{\sum_{0}^{x=i}1-\left(r_{x}/r_{0}\right)}{i}$
Radius loss Standard Deviation (std)	$s_{RL}$	$s_{RL}=\frac{\sum_{0}^{x=i}{\left(1-\left(r_{x}/r_{0}\right)-\bar{RL}\right)}^{2}}{i-1}$
Pitting Factor [36]	PF	$PF=\frac{\text{deepest metal penetration }}{\text{average metal penetration}}$
Max. pit depth (μm)	$d_{max}$	Maximum depth of all detected pits: $max\left(d_{x}\right)$
Av. of ten deepest pits (μm)	${\bar{d}}_{10}$	${\bar{d}}_{10}=\frac{\sum_{0}^{x=10}d_{x}}{10}$
Av. pit depth (μm)	$\bar{d}$	$\bar{d}=\frac{\sum_{0}^{x=n}d_{x}}{n}$
Pit depth Standard Deviation (std) (μm)	$s_{\bar{d}}$	$s_{\bar{d}}=\frac{\sum_{0}^{x=n}{\left(d_{x}-\bar{d}\right)}^{2}}{n-1}$
Av. pit opening (μm^2^)	$\bar{o}$	This is the average pit opening area of all detected pits: $\bar{o}=\frac{\sum_{0}^{x=n}o_{x}}{n}$
Pit opening Standard Deviation (std) (μm^2^)	$s_{\bar{o}}$	$s_{\bar{o}}=\frac{\sum_{0}^{x=n}{\left(o_{x}-\bar{o}\right)}^{2}}{n-1}$
Av. volume pit (μm³)	$\bar{v}$	Average volume of all detected pits: $\bar{v}=\frac{\sum_{0}^{x=n}v_{x}}{n}$
Volume pit Standard Deviation (std) (μm³)	$s_{\bar{v}}$	$s_{\bar{v}}=\frac{\sum_{0}^{x=n}{\left(v_{x}-\bar{v}\right)}^{2}}{n-1}$
Minimum fitted radius	$r_{min}$	The minimum of all fitted radii in every cross section (Fig. 2(c,vii), exemplarily for one layer dashed line): $min\left(r_{x}\right)$
Minimum Mg core width	$d_{min}$	Minimum of all detected magnesium core widths (Fig. 2(c,vii), exemplarily for one layer dotted line): $min\left(d_{Mg}\right)$

## 3 Results

### 3.1 Immersion testing

**Fig. 3:**
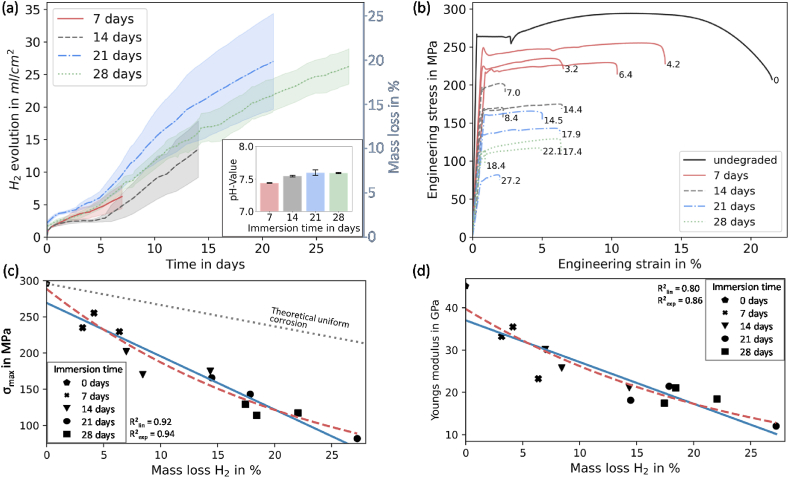
(a) Mean measured hydrogen gas evolution during immersion testing in c-SBF over time. pH values of the solution with standard deviation after immersion; (b) Tensile test data of every test specimen after specified immersion time. Number after curves is the measured mass loss calculated from hydrogen evolution. (c) Specimen strength (σ_max_) plotted against mass loss (blue solid line: linear fit, red dashed line: exponential fit, grey dotted line: theoretical uniform corrosion; p-values < 0.05) (d) Young's modulus plotted against mass loss (blue solid line: linear fit, red dashed line: exponential fit, p-values < 0.05). (For interpretation of the references to colour in this figure legend, the reader is referred to the Web version of this article.)

Fig. 3(a) shows hydrogen evolution (left axis) of the magnesium specimens over the 28-day immersion period. The corresponding mass loss of the specimens is also shown in Fig. 3 (a) (right axis). In general, the hydrogen evolution (or mass loss) rate was greatest in the first day following immersion, with the rate flattening up to day five, after which there was a secondary increase. This phenomenon can be attributed to the breakage of the protective magnesium oxide layer. The measured pH value showed only slight pH increases during the study, increasing from 7.4 ± 0.15 at day zero over 7.43 ± 0.005 after seven days, to 7.6 ± 0.04 at day 21. Within the next week, no further increase was observed, and the standard deviation even decreased. Ng et al. showed that even a pH of 8 lead to a similar hydrogen evolution response during in-vitro testing, so our measurements are valid [49].

### 3.2 Tensile testing

Fig. 3(b) shows the uniaxial stress strain response from each magnesium dog-bone specimens (corresponding mass loss plotted at the end of every curve). These results show decreasing mechanical performance as maximum stress, yield strength and strain-to-failure of the samples are reduced as corrosion progresses. Interestingly, this magnesium alloy displays a distinct upper and lower yield point, with substantial yield elongation, likely a result of a Lüders front forming in the alloy under tension [50]. Plotting specimen strength (σ_max_) as a function of mass loss in Fig. 3(c), an exponential and linear fit is possible, but the mechanical integrity of the specimens is substantially reduced. For example, at approx. 15% mass loss the strength has reduced by approx. 50%. To underline the disproportionate relationship the theoretical uniform corrosion behaviour is included in the figure (dotted grey line). Theoretical uniform corrosion is derived from the assumption that with a material loss of 50% the remaining specimen strength is 50% from the initial strength. Fig. 3(d) shows the reduction of the stiffness with increasing mass loss.

### 3.3 Micro computed tomography

**Fig. 4:**
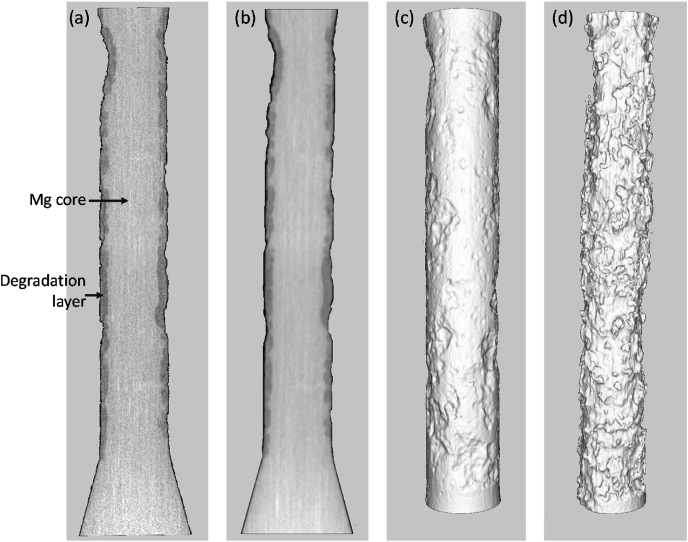
μCT scan process chain of a 14 days immersed dog bone in SBF (a) raw input cross section, darker area degradation layer, lighter area inner magnesium core (b) Gaussian filter stddev = 2.0 pixel (standard deviation of the Gaussian distribution) (c) Segmentation of the complete gauge length of dog bone (d) segmentation of inner magnesium core only.

**Fig. 5:**
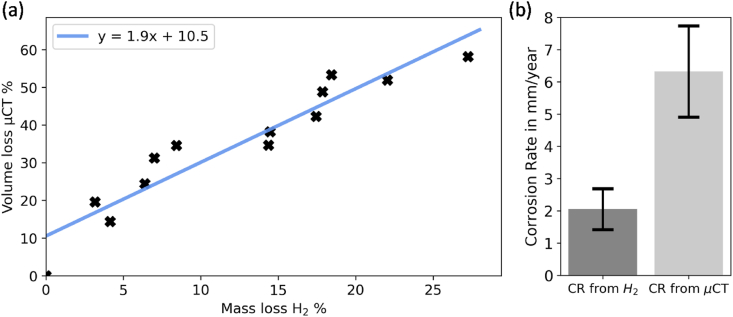
(a) Correlation measured volume loss of μCT scans to the calculated mass loss from hydrogen evolution; (b) Mean corrosion rate (CR) calculated from hydrogen evolution and evaluation of μCT-scans.

Fig. 4 shows the processing of one of the dog-bone samples, after 14 days immersion. Segmentation of the inner Magnesium core and outer degradation layer was determined using Imalytics. Fig. 5(a) shows the correlation between the mass loss from hydrogen evolution and volume loss determined through micro-CT. Fig. 5(b) shows the yearly corrosion rate calculated through ASTMG31 [44], using both methods, with the rate determined through volume loss three-fold higher than the rate calculated from the hydrogen measurement method.

### 3.4 Pit detection

**Fig. 6:**
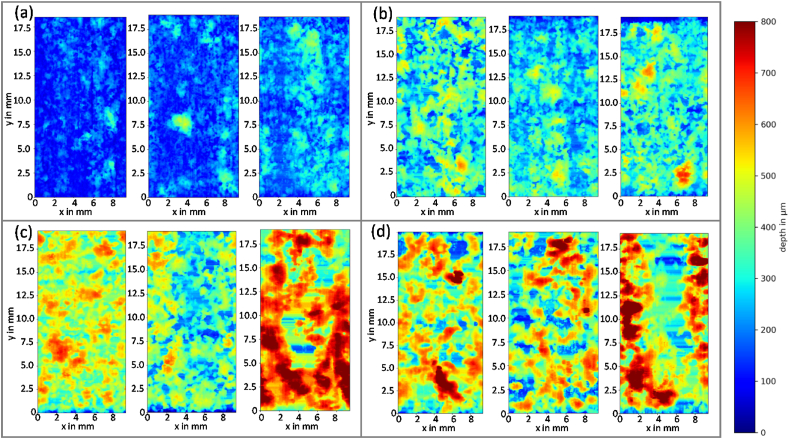
Heat plot of the measured distance from the initial radius to the surface of the Magnesium core of every tested dog bone; a) 7 days; b) 14 days; c) 21 days; d) 28 days.

**Fig. 7:**
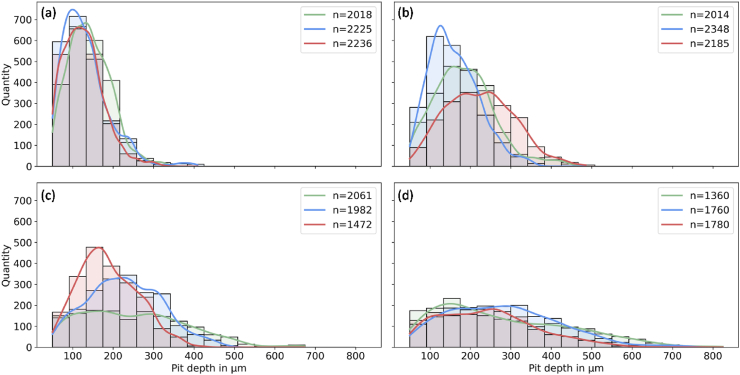
Pit depth distribution for every tested sample with n the total number of detected pits (a) 7 days; (b) 14 day; (c) 21 days; (d) 28 days.

*PitScan* provides a three-dimensional reconstruction of the processed μCT scans, along the entire gauge length of the dog bone specimens. Fig. 6 shows contour plots which describe the spatial distribution of pit depth on the (flattened) surface of the gauge section of each cylindrical specimen. Here, the contour represents the radial distance from the surface of the Mg core to the initial radius of the gauge section. Fig. 7 shows the probability distribution of pit depths (calculated by Algorithm 2) for each specimen. While Fig. 6 shows the total depth, which is the loss of material from the original surface, Fig. 7 shows the quantity of the actual pit depth compared to the current corroding surface (e.g. uniform corrosion was subtracted). At day 7, pit depths up to 400 μm are measured and there is a general increase in pit depth over time. At day 21, there is substantial localised material loss visible in certain specimens. By day 28, pit depths of over 800 μm are visible in each specimen examined and there is a general flattening of the pitting distribution visible (Fig. 7(d)), which suggest that many smaller pits develop first and these coalesce in deeper pits over time.

### 3.5 Regression fitting

**Fig. 8:**
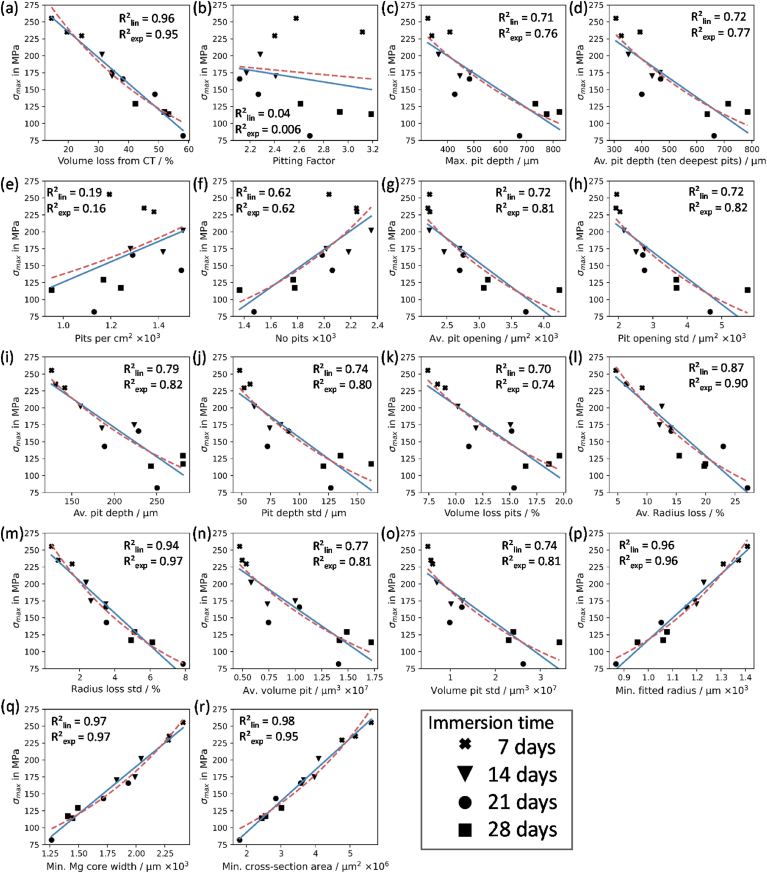
Correlation of 18 pitting features vs. max. specimen strength (σ_max_) (a–r); blue solid line: linear correlation, red dashed line: exponential correlation; p-values < 0.05 except for (b) pitting factor, (e) Pits per cm.^2.^ (For interpretation of the references to colour in this figure legend, the reader is referred to the Web version of this article.)

The main advantage of the developed method is the automatic and systematic generation of different geometrical parameters, which describe the phenomenology of pitting formation. Fig. 8 presents correlations of a range of geometric parameters that describe pitting corrosion to the specimen strength of the samples. Here, Fig. 8(a) shows again a disproportionality between the detected volume loss through micro-CT scanning and σ_max_. This relation is also tracked with the detected mass loss through the hydrogen gas measurement (Fig. 3(c)). But since the volume loss measurements are much higher than mass loss the disproportionality is not that severe. Further, a linear and exponential correlation is visible ($R_{lin}^{2}=0.96$ and $R_{exp}^{2}=0.95$).

The parameters described in ASTM G46-94 [36] are included in the examination matrix (Fig. 8(b–h)). The lowest correlation to the sample strength was found for pitting factor (Fig. 8(b)). Here the coefficient of determination for the linear and exponential fits are lower than 0.04. The generated pitting values vary from 2.1 to 3.2 but no systematic correlation was evident. Surprisingly, it was found that the strength was higher with increasing number of pits and pit density (Fig. 8 (e,f)). This relationship underlines the tracked behaviour of the pit formation in Fig. 7 showing initial high values of single pits which merge over time to form bigger and deeper pits. Looking at the two pit features regarding pit depth, suggested in the standard (max. pit depth and the average of the ten deepest pits), trends of a linear or exponential correlation can be found (Fig. 8 (c,d)). Though, the average pit depth and the average opening area of a pit with the associated standard deviations show slightly better results (R^2^ between 0.72 and 0.83) (Fig. 8(g–j)). The highest correlating pit features (both linear and exponential) are the parameters that are directly linked to the reduction of the cross-sectional area (Fig. 8 (l,m,p-r))

## 4 Discussion

In this study, a three-dimensional automated detection framework that systematically evaluates the severity and phenomenology of pitting corrosion was developed. This approach used a python-based algorithm that automatically computed geometric features of pitting from micro-CT scans of cylindrical shape specimens undergoing corrosion. Furthermore, this framework automatically outputs standardised measures of pitting corrosion on the specimen surface, including pit density, pit size, pit depth, and pitting factor (ASTM G46-94 [36]). *PitScan* was used to evaluate pitting formation in cylindrical tensile specimens of a Magnesium WE43 alloy and several relationships between pitting parameters and mechanical performance were determined. Interestingly, it was found that several of the parameters described in ASTM G46-94 showed little correlation to mechanical performance. However, several other parameters were found to show strong correlations with σ_max_ and these tended to be directly linked to the reduction of the cross-sectional area of the specimen. Specifically, our results indicate that minimum Magnesium core width and the average fitted radius over all layers (including standard deviation) are parameters that are most suited to provide an indication of a specimen's mechanical performance. *PitScan*, which was developed in this study has the potential to provide a basis to standardise measurements of pitting corrosion across a range of metals. Testing conditions like considering depths only greater than 50 μm as pits and the reduction of uniform corrosion by implementing a material ratio of 20%, were equally good applicable for samples with little and much material loss. However, it must be noted that changing those parameters would have a significant influence on the calculated features. Moreover, a reliable mechanical strength prediction is possible in the future, by investigation more tensile test specimens.

Rapid mechanical deterioration of magnesium-based medical implants has limited their implementation in load-bearing applications [8,9]. While the accelerated loss of mechanical integrity has previously been linked to pitting corrosion [17,18], our study is the first to establish quantitative relationships between key phenomenological parameters of the pit formation and the mechanical performance of medical-grade magnesium. Here, we clearly demonstrate the reduction in ultimate tensile strength for a WE43 magnesium alloy undergoing corrosion which is directly linked to pitting formation and is always the predominant corrosion mechanism in all Magnesium alloys. However, our results demonstrate that several parameters described in ASTM G46-94 provide little insight into the mechanical integrity of specimens undergoing corrosion. In particular, pitting factor showed poor correlations with ultimate tensile strength. Pitting factor describes the non-uniformity of pitting on the surface, with values of 1 corresponding to uniform corrosion. However, in scenarios with lots of similar deep pits, pitting factor does not sufficiently describe pitting formation, because it would be approximately 1 which would be an indicator for uniform corrosion, even though similar deep pits could exist. Our results also demonstrate that pit number and pit density are actually negatively correlated with reductions in ultimate tensile strength of specimens. While this may appear counter-intuitive, the detailed information provided by *PitScan* shows that many small pits are formed early on in the corrosion process, which eventually coalesce into one another over time to form larger pits (e.g. histograms in Fig. 7) that are more detrimental to load-bearing capacity. As such, it was found that parameters that are linked to maximum pit dimensions or specimen's minimum cross-sectional area better represented mechanical integrity. Therefore, pit features such as average radius loss, the minimum fitted radius and the minimum core width are potentially the best candidates for predictors of the mechanical strength (see Fig. 8). It was also observed that variabilities of factors, represented by the associated standard deviations, should be considered as candidates.

The results of this study demonstrate the novel functionality of the automated detection framework that has been developed, which enables three-dimensional systematic evaluation of surface-based pitting formation in cylindrical specimens undergoing corrosion. To date, the vast majority of studies of magnesium alloy corrosion only consider bulk measurements of material loss by monitoring hydrogen gas evolution or mass/volume loss [17,18,21,25,[27], [28], [29], [30], [31]]. When studies consider local pitting formation, they generally rely on techniques where visual inspection of surfaces and/or cross-sections are conducted [6,14,22,51,52]. This has meant that standardised measurements of pitting have been largely qualitative, two-dimensional and may require destructive processes. While certain studies have used micro-CT based approaches to analyse pitting corrosion [6,14,22,51,52], *PitScan* has the distinct advantage of providing quantitative measures through non-destructive means, with the capacity to provide complete spatial reconstruction of test specimens, which enables a complete, reproducible investigation of the corrosion progress. This framework could easily be applied to other metallic alloys undergoing corrosion and could be adapted to evaluate corrosion in more complex geometries.

Certain limitations of the study must be noted. In-vitro testing was performed according to ASTMG31 - 12a [44], to determine to corrosion process of the Magnesium alloy for different time steps with its mechanical integrity. While this does not fully represent the conditions of medical implants undergoing corrosion in-vivo, it does provide insight into the correlation between certain pit formations and the remaining mechanical strength. Additionally, the more aggressive in-vitro environment is helpful to observe earlier specific characteristics like pitting corrosion. Further testing could be carried out to provide a broader dataset to establish accurate trends for magnesium corrosion over wider timescales (e.g. mass loss percentages). It is also worth noting that, in this study, there was a three-fold difference between the tracked mass loss calculated from hydrogen evolution and the actual detected volume loss from micro-CT analysis. Liu et al. demonstrated similar differences between the corrosion rates calculated from hydrogen evolution and from micro-CT scans for different magnesium alloys [51]. They reported the corrosion rates (from volume loss and mass loss) for pure Magnesium immersed in Hank's Balanced Salt Solution (HBSS) for 14 days. They measured 0.64 mm/year for the hydrogen gas measurement (H_2_) and 1.14 mm/year for the volume loss (micro-CT), which leads to a ratio of 1.8. In general, pure magnesium degrades more slowly than its alloys [31,51,53]. In our study, we measured a mean degradation rate of 2.0 mm/year (H_2_) and 6.5 mm/years (micro-CT), leading to a ratio of 3.2. The ratio difference could be caused by the different material itself, immersion time and immersion solution. The lower detected mass loss values can be explained by the fact that until now the amount of the reduction of dissolved oxygen during the corrosion process is not fully understood, and hydrogen is also built into the degradation layer [54]. Further, the collection of hydrogen is susceptible to errors, like the formation of bubbles on the funnel, diffusion into solution, or diffusion through the equipment [45]. All this must be considered while comparing the two different approaches for measuring the material loss. Micro-CT scanning seem to be more reliable in terms of identifying the overall volume loss. However, this is associated with high costs and greater effort.

## 5 Conclusions

This study presented for the first time a three-dimensional automated detection framework that systematically evaluates the spatial progression of pitting corrosion from micro-CT scans of metallic specimens (*PitScan*). This framework is non-destructive and automatically determines a wide range of geometric measures of pitting corrosion on the specimen surface according to ASTM G46-94 [36]. By conducting mechanical tests of magnesium alloy specimens undergoing corrosion, it was found that several of the parameters (e.g. pitting factor, no. of pits) described in ASTM G46-94 showed little correlation to mechanical performance. However, several other parameters (e.g. radius loss, minimum core width) were found to show strong correlations with the remaining specimen strength and these tended to be directly linked to the reduction of the cross-sectional area of the specimen. This framework could easily be applied to other metallic alloys undergoing corrosion and could be adapted to evaluate corrosion in more complex geometries. Further coated specimens can be investigated in terms of their corrosion formation in conjunction with the mechanical integrity.

## CRediT authorship contribution statement

**Kerstin van Gaalen:** Methodology, Investigation, Software, Writing – original draft, Visualization. **Felix Gremse:** Investigation, Resources, review. **Felix Benn:** Investigation, Conceptualization, review. **Peter E. McHugh:** Conceptualization, review. **Alexander Kopp:** Conceptualization, review, Supervision. **Ted J. Vaughan:** Conceptualization, Writing - re-view & editing, Funding acquisition, Supervision.

## Declaration of competing interest

The authors declare that they have no known competing financial interests or personal relationships that could have appeared to influence the work reported in this paper.

## Figures and Tables

**Fig. 9:**
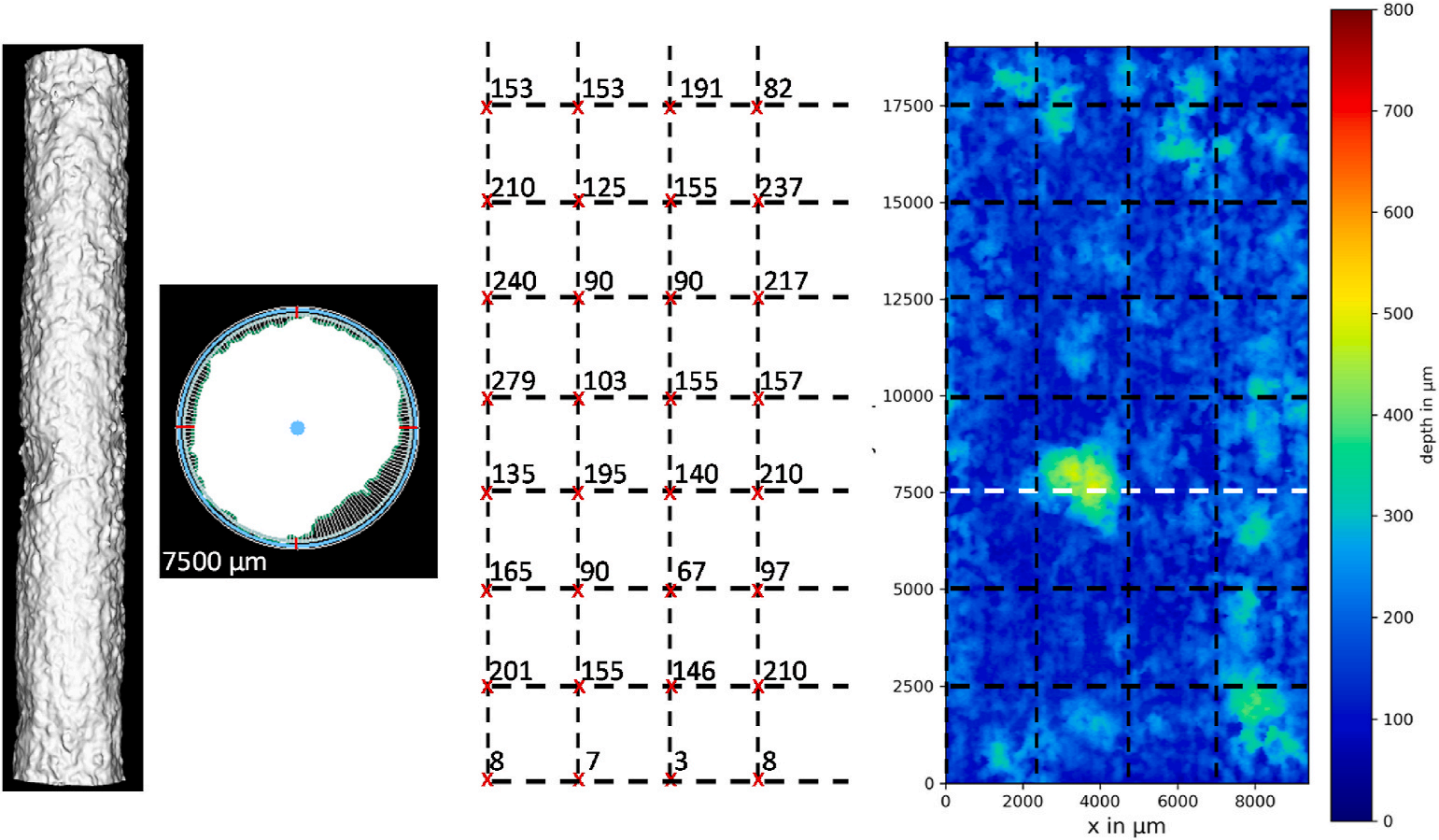
7 days sample 2.

**Fig. 10:**
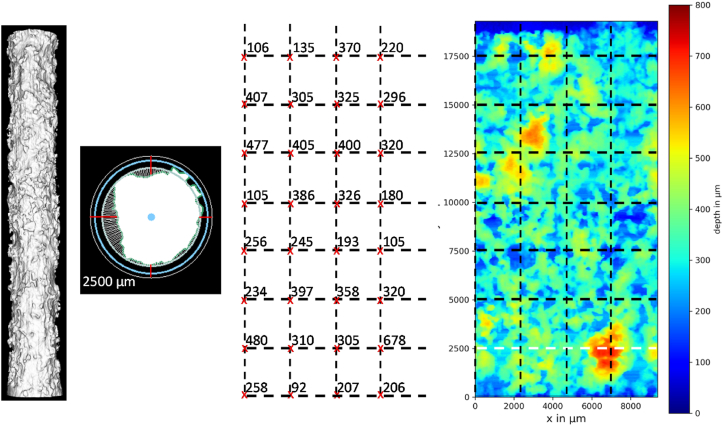
14 days sample 3.

**Fig. 11:**
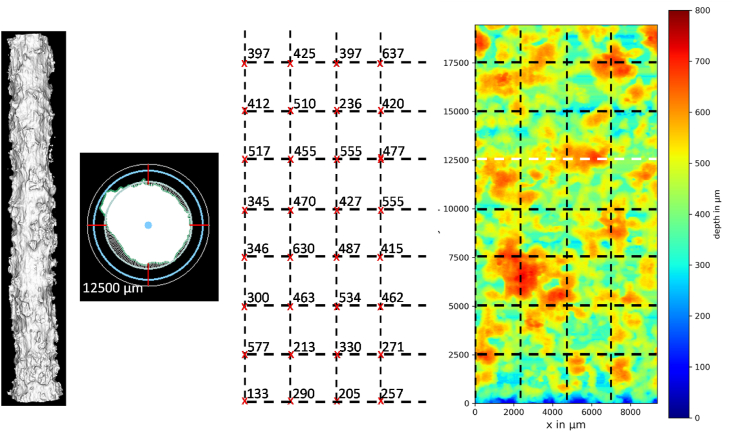
21 days sample 1.

**Fig. 12:**
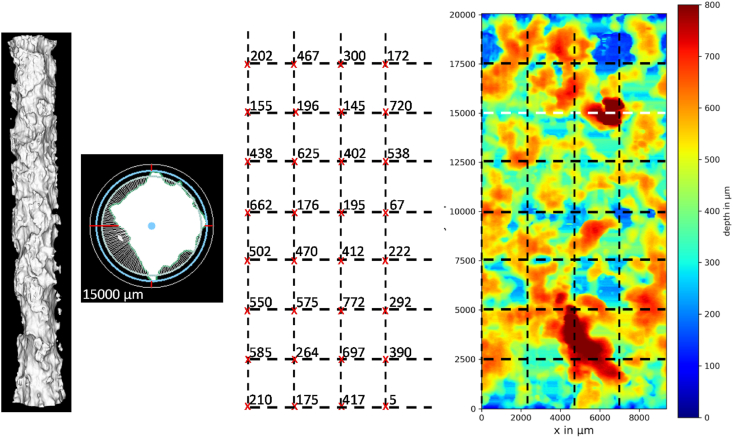
28 days sample 1.
